# Starter cultures as biocontrol strategy to prevent *Brettanomyces bruxellensis* proliferation in wine

**DOI:** 10.1007/s00253-017-8666-x

**Published:** 2017-11-30

**Authors:** Carmen Berbegal, Giuseppe Spano, Mariagiovanna Fragasso, Francesco Grieco, Pasquale Russo, Vittorio Capozzi

**Affiliations:** 1 Department of Agriculture, Food and Environment Sciences, University of Foggia, Via Napoli 25, 471122 Foggia, Italy; 2 Enolab. ERI BioTecMed, Universitat de València, 46100 Valencia, Spain; 3 Cereal Research Centre, Council for Agricultural Research and Economics, 71122 Foggia, Italy; 4 Promis Biotech srl, Via Napoli 25, 71122 Foggia, Italy; 5 Istituto di Scienze delle Produzioni Alimentari, Consiglio Nazionale delle Ricerche, Unità Operativa di Supporto di Lecce, Lecce, Italy

**Keywords:** *Brettanomyces bruxellensis*, Wine, *Saccharomyces*, malolactic fermentation (MLF), Lactic acid bacteria

## Abstract

*Brettanomyces bruxellensis* is a common and significant wine spoilage microorganism. *B. bruxellensis* strains generally detain the molecular basis to produce compounds that are detrimental for the organoleptic quality of the wine, including some classes of volatile phenols that derive from the sequential bioconversion of specific hydroxycinnamic acids such as ferulate and *p*-coumarate. Although *B. bruxellensis* can be detected at any stage of the winemaking process, it is typically isolated at the end of the alcoholic fermentation (AF), before the staring of the spontaneous malolactic fermentation (MLF) or during barrel aging. For this reason, the endemic diffusion of *B. bruxellensis* leads to consistent economic losses in the wine industry. Considering the interest in reducing sulfur dioxide use during winemaking, in recent years, biological alternatives, such as the use of tailored selected yeast and bacterial strains inoculated to promote AF and MLF, are actively sought as biocontrol agents to avoid the “Bretta” character in wines. Here, we review the importance of dedicated characterization and selection of starter cultures for AF and MLF in wine, in order to reduce or prevent both growth of *B. bruxellensis* and its production of volatile phenols in the matrix.

## Introduction

The success of winemaking in terms of safety and quality considerably depends on the metabolism of microorganisms present on the grapes and during the fermentation process (Grangeteau et al. 2017, Liu et al. 2017). Several microbial species can cause depreciation of wine since they produce detrimental compounds that negatively affect wine aroma and flavors (Suárez et al. 2007). Among the spoilage microorganisms, the yeast *Brettanomyces bruxellensis* is generally considered one of the most relevant in term of depreciation potential. This species, because of its ability to survive during the winemaking process, within several years, has become a major oenological concern worldwide (Di Toro et al. 2015, Steensels et al. 2015, Capozzi et al. 2016). This species can persist through the harsh conditions, such as ethanol concentrations associated to the alcoholic fermentation (AF) and increasing additions of sulfur dioxide (SO_2_). *Brettanomyces* strains are well suited to surviving on all surfaces, in and around the winery: winery walls, presses, and fermentation tanks as well as within the barrels used for aging (Fugelsang 1997). Furthermore, the biofilms formed by *B. bruxellensis* causes important problems, as microbial cells in biofilms often showed an increased resistance to stressing conditions, including chemical cleaning agents and sanitisers (Oelofse et al. 2008).

*Brettanomyces bruxellensis* is able to live in environments uninhabited by other microorganisms, due to the “desolation” of these media, because the simultaneous presence of different stressors, e.g., high ethanol content, low pH, and starvation (Smith and Divol 2016). The genome sequencing has revealed genes allowing for the utilization of a varied range of substrates (Curtin and Pretorius 2014, Crauwels et al. 2015). In grape must, *Saccharomyces cerevisiae* is strongly adapted and easily dominates *B. bruxellensis*. In contrast, *B. bruxellensis* is well adapted to the physico-chemical conditions characterizing wines after the AF (Nardi et al. 2010). The mechanisms regarding either the growth in wine of *B. bruxellensis* or how it can outcompete all other yeasts after AF are nowadays not fully understood.

The risk of microbial spoilage can be minimize by the application of good cellar hygienic practices such as the reduction of the latent phase between the end of AF, the good performed malolactic fermentation (MLF), and the early wine stabilization. For years, SO_2_ has been employed as chemical preservative by winemakers for its antioxidant and microbiostatic properties (Divol et al. 2012, Zuehlke et al. 2013), and it is the most commonly added preservative to grape must and wine to limit the development of *B. bruxellensis* and other unwanted microorganisms (Couto et al. 2005, Oelofse et al. 2008). The response of *B. bruxellensis* to SO_2_ has been extensively studied (Longin et al. 2016) and various surviving strategies have been reported including sulfur reduction, acetaldehyde production, active sulfur efflux, and ability of this yeast to enter in a viable but not culturable (VBNC) state (Serpaggi et al. 2012; Divol et al. 2012; Capozzi et al. 2016). During the VBNC state, the yeast cells are able to remain viable while temporarily losing their ability to proliferate on culture media (Capozzi et al. 2016). Moreover, different strains display a range of sensitivity to SO_2_ (Louw et al. 2016), also in terms of SO_2_-induced VBNC state (Capozzi et al. 2016). In addition, investigations from Curtin et al. (2011) showed that *B. bruxellensis* isolates exhibit strain-dependent tolerance to sulphite. Considering human consumption, it is important to underline how these preservative molecules are usually linked to adverse effects in wine consumers, including allergic reactions, asthma and headaches (Guerrero and Cantos-Villar 2015). Several physico-chemical approaches have been tested to avoid undesired proliferation in wine contaminated by *B. bruxellensis*, providing an overview of these applications and underlining the main pros and cons about their use in oenology (Table 1).

**Table 1:** Possible treatments for the control of *B. bruxellensis* in wine

Treatment	Benefits	Disadvantages	Reference
Heat	Destroys microorganisms	Only used in barrels	Fabrizio et al. 2015
Filtration	Reduces the number of cells by physical separation	Loss of color and aroma	Duarte et al. 2017
Protein clarification	Reduces the number of cells by flocculation	Loss of color and aroma	Murat and Dumeau 2003
SO_2_	Inhibits cell proliferation. Prevents the ethylphenols formation and oxidation	Microbial resistance. Adverse effects in human health	Guerrero and Cantos-Villar 2015
Chitosan	Inhibits cell proliferation. Prevents the ethylphenols formation	Loss of color. Only from fungal origin is permitted	Portugal et al. 2014
Dimethyl dicarbonate	Inhibits cell proliferation. Prevents the ethylphenols formation	High costs. Dosing machine is needed	Renouf et al. 2008
High pressure	Eliminates cells	High costs. Pressure and time dependent	van Wyk and Silva 2017
Pulsed electric fields	Eliminates cells	High costs.	Puertolas et al. 2009

Biological alternatives are increasingly explored, including the use of starter cultures tailored to control spoilage microorganisms (García-Moruno and Muñoz 2012, Oro et al. 2014). Since the first developments of starter cultures technology in the wine sector, a particular attention has been deserved on the exploitation of intraspecific biodiversity within species responsible for AF (*S. cerevisiae*) and for MLF (*Oenococcus oeni* and *Lactobacillus plantarum*) (Berbegal et al. 2016). Moreover, in the last decade, several studies suggested the oenological application of strains/species belonging to the heterogeneous class of non-*Saccharomyces* yeasts (de Benedictis et al. 2011; Tristezza et al. 2016b; Berbegal et al. 2017b; Petruzzi et al. 2017). These species offer new opportunities to develop biotechnological solutions to cope with specific problems, hence improving the quality and safety of wines (Petruzzi et al. 2017). The aim of this review is to draw up a record of the current knowledge on the use of tailored starter cultures against *B. bruxellensis* yeast and their application in winemaking conditions.

## Chemistry and *B. bruxellensis* development in wine: the production of off-flavors

The undesirable compounds most commonly associated with *B. bruxellensis* in wine contaminations are 4-vinylphenol, 4-vinylguaiacol, 4-ethylphenol (4-EP), and 4-ethylguaiacol (4-EG) (Chatonnet et al. 1995, Harris et al. 2008). The production of high concentrations of 4-EP are associated with unpleasant aromas described as “stable,” “horse sweat,” or “leather” (Chatonnet et al. 1995, Steensels et al. 2015). In last years, the formation of these compounds has been deeply studied and several reviews that highlight this topic have been published (Suárez et al. 2007, Wedral et al. 2010).

**Fig. 1:**
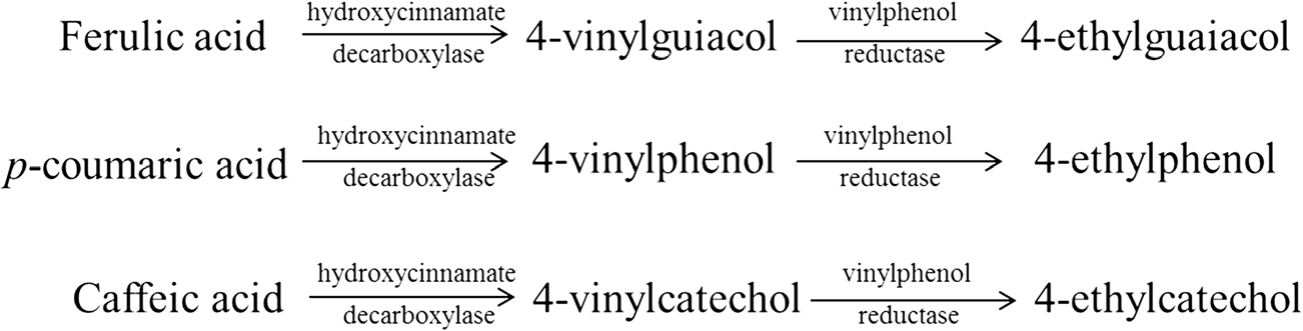
Formation of ethylphenols from their hydroxycinnamic precursors

The origin of volatile phenols involves the sequential action of two enzymes on a hydroxycinnamic acid (ferulic, p-coumaric, or caffeic acid) substrate. In the first step, the hydroxycinnamate decarboxylase transforms the hydroxycinnamic acids into vinylphenols (Edlin et al. 1998), and then, the vinylphenol reductase reduced them to ethyl derivatives (Dias et al. 2003) (Fig. 1).

Recent studies have demonstrated that *B. bruxellensis* is not the only microorganism that can produce 4-EP and 4-EG and that the capacity to produce these compounds is a strain-specific feature (Conterno et al. 2010). Several other microorganisms, including some lactic acid bacteria (LAB) and non-*Saccharomyces* yeasts, are able to produce volatile phenols (Chatonnet et al. 1995, Fras et al. 2014). What differentiates *B. bruxellensis* from the other microorganisms is its capacity to synthetize the highest amounts of these ethylphenols (Dias et al. 2003, Malfeito-Ferreira 2011). Different concentrations of 4-EP and 4-EG appear in wine depending on the variety of grape used, vinicultural conditions, and winemaking practices (Wedral et al. 2010). 4-EG are associated with descriptive expressions such as “bacon” or “smoked” (Suárez et al. 2007). Another ethylphenol produced by *B. bruxellensis* is the 4-ethylcatechol (4-EC), which has the caffeic acid as precursor and it is denoted by its medicinal aroma. 4-EC has, usually, a lower detection threshold than other ethyl phenols (Loureiro and Malfeito-Ferreira 2006). *B. bruxellensis* can metabolize only the free-form of *p*-coumaric, caffeic, and ferulic acids. Therefore, the conversion of coutaric acid by the cinnamyl esterase enzyme to *p*-coumaric acid by other microorganisms can contribute to the increased production of ethyl phenols by *B. bruxellensis* (Schopp et al. 2013).

## Controlling volatile phenol formation

### Preventing the increase of the concentrations of precursors of volatile phenols

**Fig. 2:**
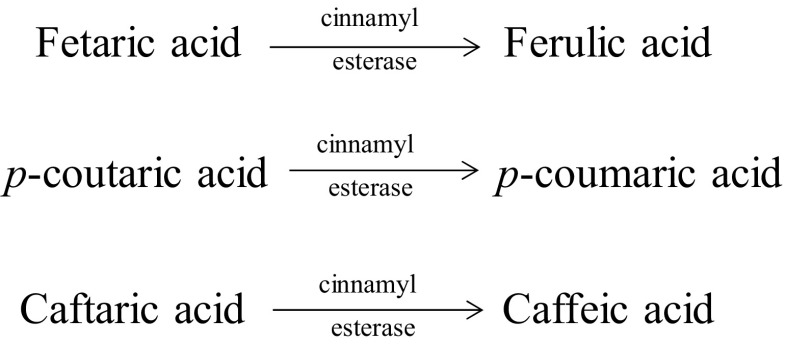
Formation of free hydroxycinnamic acids from their esters of tartaric acid precursors

Ferulic, *p*-coumaric, and caffeic acids are naturally present in grape must and are typically found as esters of tartaric acid (fetaric, coutaric, and caftaric acids, respectively). During winemaking, these tartaric acid esters can be hydrolyzed, forming free hydroxycinnamic acids (Nagel and Wulf 1979). *B. bruxellensis* can metabolize only the free-form of these hydroxycinnamic acids. Therefore, the conversion of, for example, coutaric acid by the cinnamyl esterase enzyme to *p*-coumaric acid by other microorganisms as LAB can contribute to the increased production of ethylphenols by *B. bruxellensis* by increasing the concentration of ethylphenol precursors (Schopp et al. 2013) (Fig. 2).

A possible strategy to reduce the precursors of ethylphenols is the use of *S. cerevisiae* strains with hydroxycinnamate decarboxylase (HCDC+) activity and able to carry out the AF (Suárez-Lepe and Morata 2012). The vinilphenols formed are able to spontaneously react with grape anthocyanins producing vinylphenolic pyranoanthocyanins. These molecules are stable pigments under oenological conditions, which can reduce the concentration of ethylphenol precursors (Romero and Bakker 2000; Bakker and Timberlake 1997). Morata et al. (2013) fermented grape musts using HCDC+ yeast strains, previously treated with cinnamylesterases in order to quickly release the grape hydroxycinnamic acids. The treated musts showed lower contents of 4-EP than those fermented by employing HCDC strains. The reduction in the ethylphenol content was due to the transformation of hydroxycinnamic acids in stable vinylphenolic pyranoanthocyanins pigments (Morata et al. 2013).

Studies from Hernández et al. (2007) and Cabrita et al. (2008) demonstarted that an increase in free hydroxycinnamic acids concentrations in wine at the end of the MLF was recorded. Nevertheless, Burns and Osborne (2013) observed an increase in *p*-coumaric and caffeic acids after MLF, and in this case, the fermentation was carried out by a commercial *O. oeni* strain. Chescheir et al. (2015) examined 10 commercial *O. oeni* strains for their ability to degrade tartaric acids—hydroxycinnamic acids—in Pinot noir wine. All strains completed MLF but one strain was able to degrade the caftaric and coutaric acids, thus increasing the amounts of caffeic and *p*-coumaric acids (Chescheir et al. 2015). The augmented free hydroxycinnamic acid content in wines significantly increased the production of 4-EP and 4-EG during growth of an inoculated *B. bruxellensis* strain. These studies confirm the importance of the inoculation of appropriately selected strains of *S. cerevisiae* and LAB to carry out the AF and MLF in order to control the volatile phenol precursors.

Performing spontaneous MLF increases the spoilage potential of *B. bruxellensis* in wine. Indeed, indigenous wine LAB associated with MLF may be able to degrade tartaric acid–hydroxycinnamic acids. Therefore, the selection criteria for commercial malolactic starters include the inability to degrade tartaric acid—hydroxycinnamic acids—in order to ensure satisfactory organoleptic properties of the final wine. Additional research to identify the genes encoding the *O. oeni* tartaric acid—hydroxycinnamic acid esterase—would enable a more efficient selection of wine LAB strains usable as commercial cultures.

### Preventing the volatile phenol formation by lactic acid bacteria

Even though *B. bruxellensis* is not the only microorganism able to produce significant amounts of ethylphenols (Chatonnet et al. 1992), other microbes are capable to synthetize volatile phenols. Some LAB, such as *Pediococcus* and *Lactobacillus* are also able to produce volatile phenols from free hydroxycinnamic acid as *p*-coumaric, caffeic, and ferulic acids (Couto et al. 2006, Fras et al. 2014). For instance, *Lactobacillus brevis* and *Pediococcus pentosaceus* are able to produce significant amounts of 4-VP, but only traces of ethylphenols. *L. plantarum* is the only bacteria able to produce significant amounts of 4-EP (Chatonnet et al. 1995). Madsen et al. (2016) investigated the effect of two commercial *O. oeni* strains, with or without cinnamoyl esterase activity, on the contents of the hydroxycinnamic acids (p-coumaric and ferulic acid) in wine. Moreover, the authors studied the formation of volatile phenols 4-ethylphenol and 4-ethylguaiacol during a period of 6 months in Cabernet Sauvignon wines inoculated with two different *B. bruxellensis* strains. The authors suggested that the level of volatile phenols in wine was mainly associated with *B. bruxellensis* strain rather than the cinnamoyl esterase activity of *O. oeni* (Madsen et al. 2016).

Couto et al. (2006) studied the ability of 35 different strains of LAB to produce volatile phenols in culture medium. Results showed that 37% of the strains were capable of producing volatile phenols from *p*-coumaric acid, and that 9% could produce 4-EP. Chatonnet et al. (1997) studied the influence of polyphenolic compounds on the production of volatile phenols by LAB and found that tannins affect either the *L. plantarum* growth or the phenolic compound production, although synthesis of volatile phenols by *B. bruxellensis* was unaffected.

In order to avoid the formation of volatile phenols by LAB, a preventive approach is to carry out a safe and controlled MLF, by using commercial starters unable to form these undesirable compounds. However, the induction of MLF by commercial starters is not always successful because wine is a very harsh environment (Ruiz et al. 2010). The employment of autochthonous starter cultures that are well adapted to the conditions of a specific wine-producing area has been suggested (Ruiz et al. 2010). This feature may represent a concrete opportunity, if we consider that a huge number of studies have been performed on the characterization of autochthonous *O. oeni* and *L. plantarum* associated to spontaneous MLF in regional wines (Garofalo et al. 2015, Sun et al. 2016, Berbegal et al. 2016, Berbegal et al. 2017a, Brizuela et al. 2017).

### Inhibiting *Brettanomyces bruxellensis* growth using non-*Saccharomyces* yeasts

The world wine market has an increase interest in new yeast strains with novel properties (Mylona et al. 2016, Petruzzi et al. 2017). Numerous studies on the influence of non-*Saccharomyces* yeast in winemaking have highlighted the oenological and technological relevance of these yeast species (Comitini et al. 2011, Tristezza et al. 2016b). Recently, some commercial yeast manufacturers have already included non-*Saccharomyces* yeast starters in their oenological products (Petruzzi et al. 2017). Strains of non-*Saccharomyces* yeasts have also shown potential for producing killer toxins with a broader spectrum of activity, inhibiting species within the non-*Saccharomyces* and the *Saccharomyces* genera (Petruzzi et al. 2017). Killer yeast strains have the characteristic of secreting toxins of proteinaceous nature that are lethal to sensitive yeast cells. The killer phenomenon in yeasts was first discovered in *S. cerevisiae* (Bevan and Makower 1963) and, then, reported to be present in many other yeast genera or species (Marquina et al. 2002, Liu et al. 2017).

Since the first record of a killer toxin inhibiting an apiculate yeast (Ciani and Fatichenti 2001), several studies focusing on yeast killer toxins have been conducted with the aim to contrast spoilage wine yeasts such as *B. bruxellensis*. Mehlomakulu et al. (2014) identified from the wine yeast *Candida pyralidae* two killer toxins, CpKT1 and CpKT2, that showed to possess a specific killer activity against several *B. bruxellensis* strains. A similar action was described for the killer toxins isolated from *T. delbrueckii* (Villalba et al. 2016), *Ustilago maydis* (Santos et al. 2011), *Klyveromyces wickerhami* and *Pichia anomala* (Comitini et al. 2004), and *Pichia membranifaciens* (Belda et al. 2017) (Table 2). These killer toxins were both active at oenological conditions, confirming their potential use as a biocontrol tool in winemaking process. Under winemaking conditions, the killer toxin Kwkt was efficient and comparable to the use of SO_2_ in inhibiting *B. bruxellensis* (Comitini and Ciani 2011). Killer toxins Kwkt and Pikt maintain their killer activity for 10 days in wine (Comitini et al. 2004). The killer toxins active against *B. bruxellensis* were stable at acidic pH ranges and at temperatures between 20 and 25 °C, which were compatible with winemaking conditions. Besides, these killer toxins were applied in trial fermentations without affecting the population of *S. cerevisiae* (Santos et al. 2009, Comitini and Ciani 2011, Santos et al. 2011). In addition, the metabolic by-products ethyl acetate and 4-ethylphenol were not detected and volatile acidity was reduced, confirming the antimicrobial efficiency of these killer toxins (Comitini and Ciani 2011, Santos et al. 2011).

**Table 2:** Killer toxins secreted by non-*Saccharomyces* yeast against *B. bruxellensis* that have potential application in wine industry

Yeast/filamentous fungus specie	Killer toxin	Mode of action	Reference
*Kluyveromyces wickerhamii*	Kwkt	–	(Comitini and Ciani 2011)
*Pichia anomala*	Pikt	–	(Comitini et al. 2004)
*Pichia membranifaciens*	PMTK2	Cell cycle arrest/apoptosis	(Belda et al. 2017)
*Candida pyralidae*	CpKT1	Cell Wall and membrane disruption	(Mehlomakulu et al. 2014)
*Candida pyralidae*	CpKT2	–	(Mehlomakulu et al. 2014)
*Ustilago maydis*	KP6	K+ depletion	(Santos et al. 2011)
*Torulospora delbrueckii*	TdKT	Cell wall disruption and apoptotic death processes	(Villalba et al. 2016)

Other biological methods to control *B. bruxellensis* using non-*Saccharomyces*-specific strains have been recently investigated. For example, Oro et al. (2014) showed that *Metschnikowia pulcherrima* secretes pulcherriminic acid, which is an inhibitory to the growth of *B. bruxellensis*. Moreover, cell-to-cell contact and quorum sensing have been investigated as mechanisms involved in non-*Saccharomyces*-mixed fermentation. In this regard, quorum sensing was recently examined in *H. uvarum*, *Torulaspora pretoriensis*, *Zygosaccharomyces bailii*, *Candida zemplinina*, and *B. bruxellensis*. Results indicated species-specific kinetics for the production of 2-phenylethanol, tryptophol, and tyrosol, considered the main molecules involved in the quorum sensing mechanism (Zupan et al. 2013, Avbelj et al. 2016).

### Inhibiting *Brettanomyces bruxellensis* growth using malolactic starters

Using selected yeasts and an appropriate yeast nutrition, winemakers safeguard a rapid, effective, and complete AF, which prevents the development of spoilage microorganisms (Abrahamse and Bartowsky 2012). However, one of the critical points during the winemaking process in which undesired microorganisms such as *B. bruxellensis* can develop is the period ranging from the end of AF to the start of MLF. At this stage, there are still some nutrients available to the spoilage microorganisms and, at the same time, microbial competitors are missing, considering that the indigenous LAB consortium is not yet established. Early inoculation with LAB after AF has been suggested as a useful way to control the proliferation of *B. bruxellensis*. Investigations from Gerbaux et al. (2009) showed that MLF began much sooner in Pinot Noir wines inoculated with two different wine bacteria, which contributed to a shorter duration for the winemaking process and significantly reduced the concentrations of volatile phenols (Gerbaux et al. 2009). Moreover, the inoculation of selected wine bacteria at the beginning of the AF is a solution to shorten the time-lapse between AF and MLF and thereby prevent the development of *B. bruxellensis*. Yeast and bacteria co-inoculation permits a reduction in overall vinification time and this is generally advantageous to the winery from an economical perspective (Abrahamse and Bartowsky 2012, Cañas et al. 2015). The wine is microbiologically stable, reducing the contamination by spoilage microorganisms, and this permits an earlier addition and reduced amounts of SO_2_ (Renouf and Murat 2008, Gerbaux et al. 2009). In this case, the importance to assess a microbial-compatibility before their utilization in industrial vinification is crucial (Alexandre et al. 2004, Tristezza et al. 2016a).

Recent studies have been performed by co-inoculating yeasts with commercial LAB strains in red grape must (Abrahamse and Bartowsky 2012, Muñoz et al. 2014, Tristezza et al. 2016a). Muñoz et al. (2014) investigated the inoculation of one commercial *O. oeni* strain with two *S. cerevisiae* strains following three different inoculation strategies: simultaneous, 3 days after the yeast inoculation or when AF was close to its end. Early bacterial inoculations with each of the two yeast strains allowed for the rapid development of the bacterial populations and the MLF duration was reduced to 6 days. Abrahamse and Bartowsky (2012) and Tristezza et al. (2016a) evaluated the interactions between commercial yeast and *O. oeni* strains. Their results indicated that simultaneous yeast and bacteria inoculation at the beginning of AF reduced the duration of the process and simultaneously lowered volatile acidity. Similar results were obtained when experiments were carried out with autochthonous *O. oeni* strains co-inoculated with *S. cerevisiae* (Izquierdo Cañas et al. 2012, Cañas et al. 2015

## Conclusion

The use of starter cultures for the control of fermentative processes and production of wine with standardized quality is well recognized. Nevertheless, here, we highlighted a further role of selected cultures on (i) the control of development of the spoilage yeast *B. bruxellensis* and (ii) to prevent volatile phenol formation. Handling the winemaking process by promoting AF and MLF through selected starter cultures inoculation is a crucial point to avoid the development of spoilage microorganisms. Inoculation with selected LAB to induce and accelerate MLF has been reported to be an effective biotechnological tool able to prevent *B. bruxellensis* contamination. However, an important stage in the malolactic bacteria selection must consider their capacity to inhibit the production of free hydroxycinnamic acids without producing volatile phenols. Besides, appropriate inoculation strategies such as co-inoculation and early or sequential inoculation right after AF could be an effective approaches to prevent the development of *B. bruxellensis*.

Furthermore, investigations on non-*Saccharomyces* yeasts possibly denoted by killer yeast activity will supply interesting alternative tools for controlling *B. bruxellensis*. However, killer toxins from non-*Saccharomyces* have not yet characterized as well as those of *S. cerevisiae*, and further investigation should be performed in order to identify their genetic origin, mode of action, and how to employ them at commercial and industrial scale.
